# Determining the Effect of Centrifugal Force on the Desired Growth and Properties of PCPDTBT as *p*-Type Nanowires

**DOI:** 10.1186/s11671-017-1851-0

**Published:** 2017-01-23

**Authors:** Muhamad Doris, Fakhra Aziz, Haya Alhummiany, Tahani Bawazeer, Nourah Alsenany, Alaa Mahmoud, Rozalina Zakaria, Khaulah Sulaiman, Azzuliani Supangat

**Affiliations:** 1 Low Dimensional Materials Research Centre, Department of Physics, University of Malaya, Kuala Lumpur, 50603 Malaysia; 2 Department of Electronics, Jinnah College for Women, University of Peshawar, Peshawar, 25120 Pakistan; 3 Center of Nanotechnology, Department of Physics, Faculty of Science, King Abdulaziz University, Jeddah, Saudi Arabia; 4 Department of Chemistry, Faculty of Applied Science, Umm Al-Qura University, Makkah, Saudi Arabia; 5 Department of Physics, Faculty of Science, King Abdulaziz University, Jeddah, Saudi Arabia; 6 Photonics Research Centre, University of Malaya, Kuala Lumpur, 50603 Malaysia

**Keywords:** PCPDTBT, Nanotubes, Nanowires, Centrifugation

## Abstract

In this study, low-bandgap polymer poly{[4,4-bis(2-ethylhexyl)-cyclopenta-(2,1-*b*;3,4-*b*′)dithiophen]-2,6-diyl-*alt*-(2,1,3-benzothiadiazole)−4,7-diyl} (PCPDTBT) nanostructures have been synthesized via a hard nanoporous alumina template of centrifugal process. Centrifuge has been used to infiltrate the PCPDTBT solution into the nanoporous alumina by varying the rotational speeds. The rotational speed of centrifuge is directly proportional to the infiltration force that penetrates into the nanochannels of the template. By varying the rotational speed of centrifuge, different types of PCPDTBT nanostructures are procured. Infiltration force created during the centrifugal process has been found a dominant factor in tuning the morphological, optical, and structural properties of PCPDTBT nanostructures. The field emission scanning electron microscopy (FESEM) images proved the formation of nanotubes and nanowires. The energy-dispersive X-ray spectroscope (EDX) analysis showed that the nanostructures were composed of PCPDTBT with complete dissolution of the template.

## Background

A template-assisted method is a common technique that has been used for more than two decades to fabricate favorable nanostructures. Some nanostructures involving nanotubes, nanorods, nanowires, nanoflowers, and nanopeapods have been produced by alumina template method [1–3]. It is a versatile cost-effective procedure employed to fabricate various nanostructures using polymer solution or melt [4, 5]. The wetting process of the alumina template to fabricate nanostructures deploys infiltration of solution into the nanochannels of the template through several methods such as immersion, spin-coating, and dip-coating methods [6–8]. Employing these processes, several nanostructures have been successfully grown. For instance, nanotubes and nanorods have been produced by simple immersion and spin-coating methods [9, 10]. Apart from the fabrication processes, there are many other parameters that influence morphology of the nanostructures such as molecular weight, solution concentration, solvent properties, viscosity of polymer and immersion time, etc. [10–13]. However, some reports conclude that some of these parameters produce little effect on the fabricated nanostructures; rather, solution-template interaction plays a key role in determining morphology [14]. Moreover, it is also suggested that morphology of nanostructures produced by modified templates differ from those fabricated in commercial prototype templates [15].

The separation of particles from a surface or substratum is usually provoked by the centrifugation technique. For instance, centrifugal force is applied to detach solid materials from their concentrated suspension solution. Recently, the centrifugation technique has been used to measure the cohesion force that is developed between particles and a flat surface [16, 17]. Fundamentally, the amount of centrifugal force, which is attributed to the infiltration force, affects the morphology of the nanostructures due to augmentative quantity of infiltrated substances [17]. Some nanostructures have been fabricated using a process based on centrifugal force [5, 18].

In recent years conjugated copolymers, with alternating acceptor and donor units, have outgrown their existing polymer counterparts such as poly(3-hexylthiophene) (P3HT) due to their superior charge transport and higher efficiency potential [19]. Poly{[4,4-bis(2-ethylhexyl)-cyclopenta-(2,1-*b*;3,4-*b*′)dithiophen]-2,6-diyl-*alt*-(2,1,3-benzothiadiazole)−4,7-diyl} (PCPDTBT), a low-bandgap *p*-type conjugated copolymer from a highly interesting polymer class, is based on the donor cyclopentadithiophene and the acceptor benzothiadiazole (CPDT and BT, respectively). PCPDTBT has been recently used for organic solar cells and infrared sensor applications due to its exceptional properties [20–22]. Its significant property is high mobility of charge carrier which leads to high efficiency when employed in organic photovoltaics (OPV) [21]. To acquire the insight and then administer the underlying morphological properties of these polymers still remains an uphill task for the researchers of the present era. Many studies have been performed to alter physical properties of PCPDTBT by varying fabrication techniques and controlling ambient parameters [23–25]. Some other techniques, for instance controlling a solvent vapor annealing process to yield high crystallinity quite for opto-electronic devices, have also been adopted [19]. Despite these notable properties, comprehensive study of PCPDTBT nanostructures is still very limited apart from its application in photovoltaic, sensor, and electronic devices.

Nanostructure materials have been vastly investigated because of their unusual advantageous properties that are beyond their native nature. Electrical, optical, and structural properties are some properties that are tremendously influenced by nanostructuring these materials. Hence, in the present study, PCPDTBT has been undertaken to produce nanostructures through a unique centrifugation technique to infiltrate the solution into the nanochannels of the alumina template. A novel centrifuge method has been adopted by modifying the centrifuge tube to fabricate PCPDTBT nanostructures. This technique has revealed quite interesting morphological, optical, and structural properties of PCPDTBT that are crucial to be studied. To the best of our knowledge, fabrication of PCPDTBT nanostructures by the centrifugal process using the nanoporous alumina template in terms of the relationship between the centrifugal forces and morphologies has not yet been reported.

## Methods

**Fig. 1:**
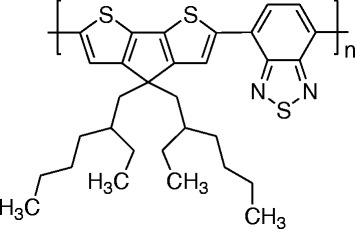
Molecular structure of PCPDTBT

Figure 1 shows the chemical structure of the conjugated polymer poly{[4,4-bis(2-ethylhexyl)-cyclopenta-(2,1-*b*;3,4-*b*′)dithiophen]-2,6-diyl-*alt*-(2,1,3-benzothiadiazole)−4,7-diyl} (PCPDTBT) that was purchased from Sigma-Aldrich and used without any further purification. Five milligrams of PCPDTBT was dissolved in 1 ml of chloroform to produce 5 mg/ml of solution, which was stirred overnight. A porous alumina template, purchased from Whatman with pore size and thickness between 20 nm and 200 nm and 60 μm, respectively, was used to fabricate nanostructures. Prior to the infiltration process, the commercial template was cleaned by immersing it into acetone and then sonicating for 15 min. Then, it was rinsed with deionized water before it was completely dried at room temperature. One hundred microliters of solution was dropped into the modified tube of centrifuge to infiltrate the channel of the cleaned alumina template. Throughout the centrifugation process, various rotational speeds of 200, 400, 800, 1000, 2000, 3000, and 4000 rpm were applied. Sodium hydroxide (NaOH) was used to dissolve the template by immersing the sample for 12 h. Prior to dissolution, the sample was stuck upside down on copper tape to flatten the remaining structures, which were further washed with deionized water for several times to completely remove the residue of NaOH. Before sample characterization, the drying process was carried out at room temperature.

**Fig. 2:**
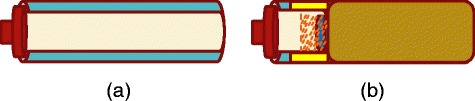
Schematic diagram of (**a**) the original tube used in the centrifugal process and (**b**) the modified tube with cantilever to maintain stability of the tube throughout the centrifugal process. (*Blue* - alumina template; *light pink* - solution; *brown* - cantilever; *yellow* - crutcher)

**Fig. 3:**
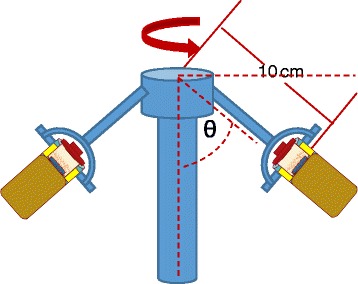
Schematic diagram for the position of the modified tube during the centrifugal process

Figure 2a, b shows the schematic diagram of the original tube (normally used in centrifugal process) and modified tube (used in this study), respectively. The centrifuge tube was modified to optimize the infiltration process of the solution into the nanochannels of the template. Modification of the centrifuge tube allows us to easily manage samples and carry out experiments properly as shown in Fig. 2. Figure 3 shows the position of the modified centrifuge tube during the centrifugal process. The length of the original tube is reduced in order to easily manage the alumina template, which is immersed into 100 μl PCPDTBT solution. Throughout the centrifugal process, the angle produced between the vertical axis and the tube is increased with increasing rotational speeds. Synthesis and characterization of samples were done using centrifuge KUBOTA 2420, field emission scanning electron microscope (FESEM-EDX) (Quanta FEG 450), UV-VIS spectroscopy (Shimadzu UV-3101PC), and Raman and photoluminescence spectroscopy (RENISHAW).

## Results and Discussion

### Derivation Related to Centrifugal Force

**Fig. 4:**
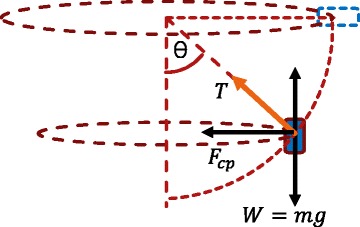
Free-body diagram of the centrifuge tube positioning during the centrifugal process and its mathematical derivation. (${F}_x=$ horizontal force, $m=$ mass, ${a}_x=$ horizontal acceleration, ${F}_{cp}=$ centripetal force, $\theta =$ angle, $\omega =$ rotational speed, $T=$ tension force, $r=$ radius and ${F}_y=$ vertical force, $g=$ gravity)

A modified centrifuge tube was designed and constructed in the present study, which is shown in Fig. 2b. The original 10-cm long centrifuge tube is modified because it uses only a small area (~5 mm^2^) of the alumina template and results in inefficient infiltration. In order to overcome this constraint and infiltrate the template effectively, a practical approach of centrifuge tube modification is realized by placing a small-sized tube within the original long tube. Besides, a cantilever is also provided to maintain stability of the smaller tube during the centrifugal process. By this approach, optimum infiltration could be achieved through higher coverage of solution onto the template. During the centrifugal process, the desired position is attained in such a way that the tube itself stays aligned with the infiltration force while the surface of the template remains parallel to the tube-base. Figure 4 illustrates the free-body diagram of the centrifuge tube position during the centrifugal process along with its mathematical derivation. Ideally, in the centrifugal process, the angle with the normal vector of the template surface should be 90° which is in line with the infiltration force vector. However, according to the tangent theta formula, the angle of 90° should equal infinity which is quite impossible to be achieved with the existing centrifuge setup. The expected angle that is acquired, here, is less than 90°. According to Newton’s First Law, in the steady state condition, the summation of horizontal forces must be equal to zero and so does the summation of the vertical forces.

1 $$ \begin{array}{l}\varSigma {F}_x=m{a}_x\\ {}{F}_{cp}=T. sin\theta \\ {}m{a}_x=T. sin\theta \\ {}m{\omega}^2r=T. sin\theta \end{array} $$

2 $$ \begin{array}{l}\varSigma {F}_y=0\\ {} mg=T. cos\theta \end{array} $$

Equation (3) is given as the relation between rotational speed and produced angle by rearranging Eqs. (1) and (2).

3 $$ \omega ={\left(\frac{g}{r} \tan \theta \right)}^2 $$

Since the centrifuge tube is placed in a fixed position during circular motion, the solution inside the tube gains pseudo force (centrifugal force) with its direction pointing out of the circular path. As shown in Eq. (4), the amount of centrifugal force is equivalent to the centripetal force which is known as the infiltration force used for permeating the porous alumina template. The calculated data of rotational speed, produced angle, and infiltration force are shown in Table 1.

**Table 1:** Data of rotational speed, produced angle, and infiltration force

Sample	Rotational speed (rpm)	Angle (θ)	Infiltration force (mN)
1	200	77.2	5.6
2	400	86.75	22.2
3	800	89.18	88.9
4	1000	89.48	138.9
5	2000	89.86	550.4
6	3000	89.94	1000.2
7	4000	89.96	2000.2

4 $$ \begin{array}{l}{F}_{cfg}={F}_{cp}\\ {}{F}_{cfg}=m{\omega}^2r\end{array} $$

**Fig. 5:**
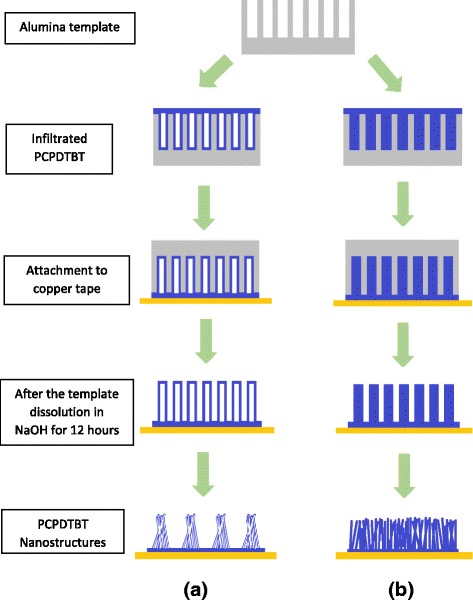
Schematic diagram of the replication of the template to form PCPDTBT (**a**) nanotubes and (**b**) nanowires

Figure 5 portrays schematic illustration of the formation of PCPDTBT nanotubes and nanowires. Firstly, the porous alumina template was cleaned by sonicating it with acetone and then rinsing with deionized water. Prior to the dissolution process of the alumina template, the cleaned template and 100 μl of PCPDTBT solution were poured into the modified tube. The whole assembly was rotated through the centrifugation process for 30 s which allowed the solution to penetrate into the nanochannels of the template. Templates with infiltrated substances were later dried under ambient room temperature. These templates were stuck upside down onto a copper tape before dissolution. The templates were dissolved using 3 M sodium hydroxide (NaOH). To wash out the alumina residue, the copper tape was rinsed several times with deionized water. Finally, the nanostructures (nanotubes or nanowires) attached to the copper tape were ready to be characterized.

### Behavior of PCPDTBT Solution due to Infiltration Force

#### Morphological Properties

PCPDTBT nanostructures have been successfully fabricated via the centrifuge method by replicating the porous alumina template. This method is a novel approach over template-wetting processes to fabricate assorted nanostructures. Wetting processes that commonly use techniques like immersion, dip-coating, and spin-coating, etc. have yielded favorable nanostructures such as nanotubes and nanorods. All these, basically, use the idea of infiltrating one solution into the cavity of the template nanochannels. However, these methods are unable to quantify the infiltration process of the solution that penetrates into the nanochannels of the template. The centrifugation method, which is proposed here, employs the wetting process onto the wall of the alumina template while quantifying the infiltration forces that permeate the nanochannels. It is a new method to correlate the infiltration forces of the solution to the morphology of produced nanostructures.

The infiltration process of filling the cavity of nanochannels can be produced by two types of forces: (1) gravitational force which employs the immersion technique and (2) capillary force that occurs on the surface of the template wall, as a consequence of a surface energy gradient between the walls of the nanochannels and the solution, when the template is dipped in a solution [8, 10]. In this study, we have fabricated nanostructures by infiltration from the centrifugation technique. The rotational speed of the centrifuge was varied with uniform time duration of 30 s. The presented novel approach helped us observe and control influence of various infiltration forces on the morphology of produced nanostructures at the same infiltration rate.

**Fig. 6:**
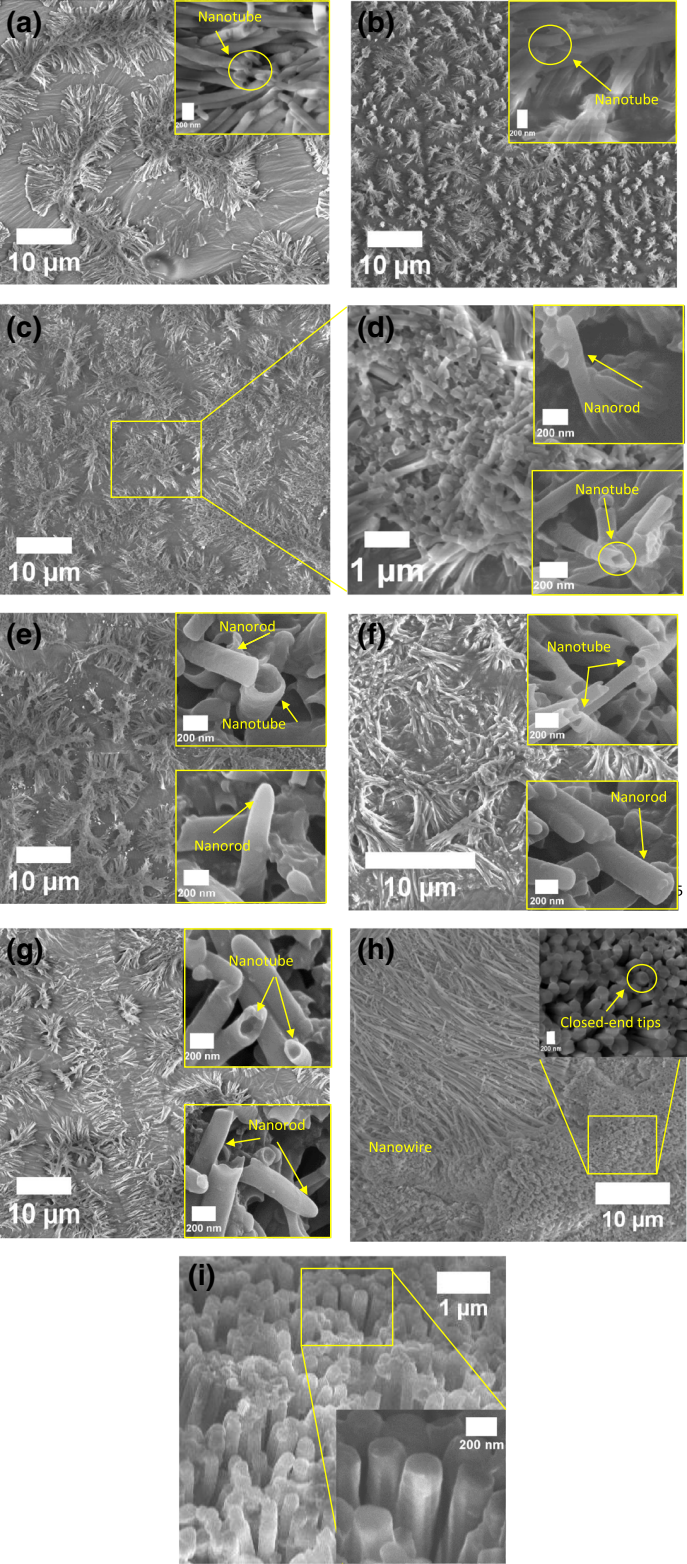
FESEM micrographs of nanostructures produced at various rotational speeds during the centrifugal process: 200 (**a**) low magnification, nanotube (*inset*); 400 (**b**) low magnification, nanotube (*inset*); 800 (**c**) low, (**d**) high magnification, nanotube and nanorod (*inset*); 1000 (**e**) low magnification, nanotube and nanorod (inset); 2000 (**f**) low magnification, nanotube and nanorod (*inset*); 3000 (**g**) low magnification, nanotube and nanorod (*inset*); 4000 (**h**) low magnification with closed-end tips (*inset*), (**i**) high magnification with free-standing nanostructures (*inset*)

Figure 6 presents the FESEM micrographs of the nanostructures at different rotational speeds during the centrifugal process. It can be clearly seen that the nanostructures are influenced by variation in the infiltration forces. Nevertheless, for all the infiltration forces, the obtained nanostructures replicate the shape of the alumina template. Once the template is dissolved by the sodium hydroxide solution, the nanostructures tend to crumple at their protruding tips and fold concurrently to form clumps of nanotubes. The FESEM results of Fig. 6 demonstrate that different rotational speeds yield both different morphologies and distributions of nanostructures. The diverse choice of infiltration forces has evidently produced an impact on shaping assorted morphologies and distribution of the produced nanostructures. The crumpling phenomena of the nanostructure tips, usually occurring during the etching process and clumping the nanotubes or nanorods into small islands, has been studied by Silverberg and co-workers as an interaction of Van der Waals between the two nanostructures [2, 3, 26–30]. At infiltration rates lower than 4000 rpm (800–3000 rpm), the nanorods are clearly distinguished from the nanotubes which can be evidenced from their tips in which the open-end tips are signed as nanotubes, meanwhile the closed-end tips are nanorods. Apparently, the transition rotational speed takes place during the process to assemble the nanostructures. At lower rotational speeds of 200–400 rpm, long-tail and tube-like structures are generally constructed that can be noticed from its hollow tips. Furthermore, at the lower forces, the gaps between the nanotube clumps are quite clearly observed.

By taking a glance at the lowest speed, i.e., 200 rpm in Fig. 6a, it can be seen that the clump size is much larger than those obtained at all higher infiltration forces with clear evidence of gaps between them. It might probably be due to small infiltration force which, however, wets the walls of the nanochannels but is not sufficient to infiltrate the entire surface of the template. At 400 rpm (Fig. 6b), the clumps are very well distributed with reduced gaps and decreased clump sizes. Here, the produced nanotubes probably meet nearly an ideal condition of the aspect ratio which leads to collapsed nanostructures because the bases of these structures are not strong enough to hold them vertically in free-standing position. Nonetheless, the tips get entangled with one another. The micrographs of Fig. 6c, e reveal a larger clump size with fairly narrow gaps between the clusters at both 800 and 1000 rpm. It can be clearly witnessed from the figure that small nanotubes and nanorods are attached at the tips of the clumps at 800 rpm as shown in the higher magnification image of Fig. 6d. However, at 1000 rpm, longer protruding tips are entangled while small nanotubes and nanorods sit atop the clumps. At these rotational speeds, the infiltration force partially fills solution into the cavity whereas some parts of the cavity remain unfilled.

Presumably, a combination of infiltration and cohesive forces has created drop-like solid nanostructures inside the nanochannels of the template which are not big enough to completely fill the cavity and form solid nanorods. Furthermore, rapid evaporation of solvent has hindered the template to be completely filled leading to the formation of nanotubes rather than nanorods. Since there exists an energy gradient between the solution and wall-surface of the template, hence, the solution spreads over the wall-surface of the nanochannels, thereby forming a thin layer of solution known as a precursor film [10, 31]. The adhesive force between the solution and the wall completely opposes the infiltration force, which gives rise to the broken nanotubes appearing on top of the clump tips. Figure 6f, g depicts that the clump size is further increased while the distance between them is reduced at 2000 and 3000 rpm. The higher infiltration force enables the solution to fill out more area of the template. Furthermore, similar to the nanostructures obtained at 800 rpm, tips of some nanotubes and nanorods slightly entangle while small nanotubes and nanorods stick to the collapsed tips. According to Fig. 6a, b, c, e, f, and g, at a lower infiltration rate, i.e., less than 4000 rpm of rotational speed, it can be observed that the nanostructures have a tendency to form a bunch of nanotubes (200–400 rpm) and a combination of nanotubes and nanorods (800–3000 rpm) that are produced during the centrifugation process.

On the contrary, at the highest infiltration force of 4000 rpm, very dense nanowires are produced which are illustrated in Fig. 6h, i with rarely entangled structures. In addition, the absence of gaps between the clumps implies that the infiltration force has homogeneously spread the solution onto the surface of the alumina template. Nevertheless, very-long-tailed nanowires with closed-end tips (inset) clearly indicate that the solution has practically fully penetrated inside the nanochannels due to higher infiltration force and produced a replica of the nanochannels of the alumina template (Fig. 6h). A few collapsed structures are also found because of a very high aspect ratio as in the case of lower infiltration forces discussed previously. These congested nanowires can possibly be employed in organic photovoltaic and sensor devices where maximum absorption of photon is required.

Below infiltration rate of 4000 rpm, the aspect ratio of a single nanotube is not suitable for free-standing stability of such nanostructures. Higher infiltration force, i.e., 4000 rpm, produces dense and free-standing nanowires due to infiltration forces far greater than adhesive forces. A less number of collapsed tips is seen at such a high infiltration rate because the high density of nanostructures does not allow the tips to be folded. Different rotational speeds of the centrifugation process have introduced different amounts of infiltration forces into the alumina template. A small amount of centrifugal force which is correlated to the infiltration force has produced various aspects of nanostructures, such as orientation, densification, and morphological distribution. It has been discerned that after the etching process applied to remove of the template, most of the nanostructures clumped with one another. An interesting feature has been noticed when a higher infiltration force ~2 N was applied; very dense nanowires with uncollapsed tips were formed. All the nanostructures possess different optical and structural properties.

**Fig. 7:**
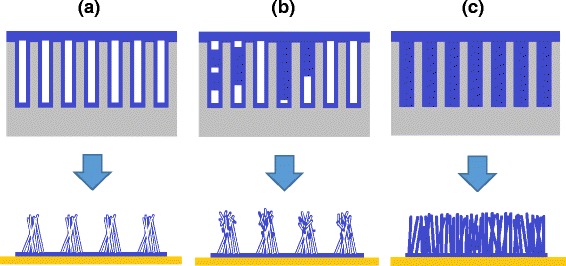
Proposed mechanism of wetting nanochannels of template and construction of (**a**) nanotubes, (**b**) partly nanotubes/nanowires, and (**c**) nanowire through infiltration forces

The proposed filling mechanism that leads to nanotubes and nanowires is shown in Fig. 7a–c. A solution that makes contact with the walls of the template nanochannels of the higher energy surface tends to spread solution over the walls due to energy gradient. During the infiltration of the solution, (a) the wetted channel walls are mostly dominated which has led to the production of nanotubes. As the infiltration force is increased, the infiltration process has only shown (b) the partially filled cavities of nanochannels with some parts remaining unfilled. At the certain saturated force, (c) the fully wetted walls and filled cavity of nanochannels are depicted.

#### EDX

**Fig. 8:**
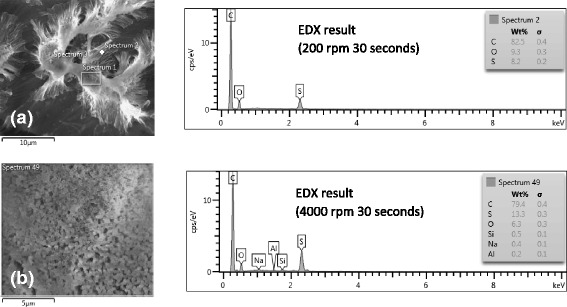
EDX results of samples that were dissolved in NaOH for 12 h with rotational speed of (**a**) 200 and (**b**) 4000 rpm

The infiltrated templates were dissolved using NaOH for 12 h. The energy-dispersive X-ray spectroscope (EDX) results show total dissolution of the alumina template as depicted in Fig. 8a, b. NaOH concentration was varied depending on the amount of substance that was used to infiltrate the template. The alumina that still remained in the slits of the nanostructures was eradicated by using an appropriate concentration of NaOH to dissolve the infiltrated templates. Based on our rigorous exercise, it was found that 1–3 M of NaOH fully dissolved the alumina leaving behind pure nanostructures adhered to the copper tape surface as evidenced from EDX patterns.

#### Optical Properties

**Fig. 9:**
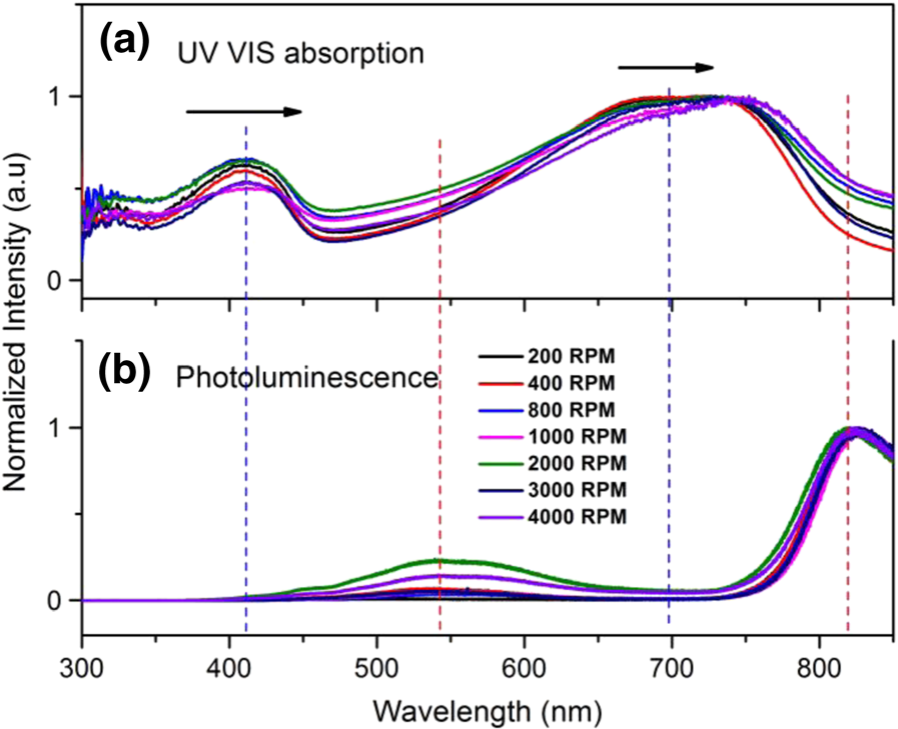
**a** Normalized UV-VIS absorption spectra of PCPDTBT. **b** Photoluminescence spectra of PCPDTBT

The UV-VIS absorption and the photoluminescence (PL) spectra of PCPDTBT are simultaneously shown in Fig. 9. The absorption spectra exhibit two main broad peaks at ~400 and ~750 nm which have been previously reported by Zhu et.al [32]. The PCPDTBT polymer chain comprises donor unit cyclopentadithiophene (CPDT) and the acceptor unit benzothiadiazole (BT) which are responsible for the absorption of shorter and longer wavelengths, respectively [21, 32].

Dissimilar infiltration forces leading to different morphology and distribution of nanostructures have produced distinct optical responses which are denoted by a peak-shift in Fig. 9a. For all the rotational speeds, the peak-shift is not significant at shorter wavelengths. However, at longer wavelengths, those nanowires fabricated at 4000 rpm exhibit a bathochromic shift (red-shift) of approximately 22 nm which corresponds to the acceptor unit (BT). The red-shifted observed for 4000 rpm, could be attributed to strong interchain interactions and elongated conjugated segments of the polymer [33]. Accordingly, a high density of nanowires might also contribute to the red-shift due to more effective polymer interchain interactions thereby absorbing lower energy which is not possible at lower infiltration forces.

It has been reported by Scharsich and co-workers that the photoluminescence spectra of PCPDTBT in solution depends upon concentration and temperature in which conformational transformation from disorder to order-state takes place at about 300 K [23]. In this study, the photoluminescence results, shown in Fig. 9b, indicate that the morphology of nanostructures does not significantly depend on photoluminescence and hence produce no remarkable shift in the peaks. The shift is only visible in Stokes which is represented by a black arrow in UV-VIS absorption of Fig. 9a. The emission spectra lie at longer wavelengths relative to the absorption wavelength at about ~550 and ~820 nm, respectively. The shift in the Stokes is produced as a result of π-π* electronic transition within the polymer chain where some of the absorbed energy is used as non-radiative energy.

#### Structural (Raman Shifting)

**Fig. 10:**
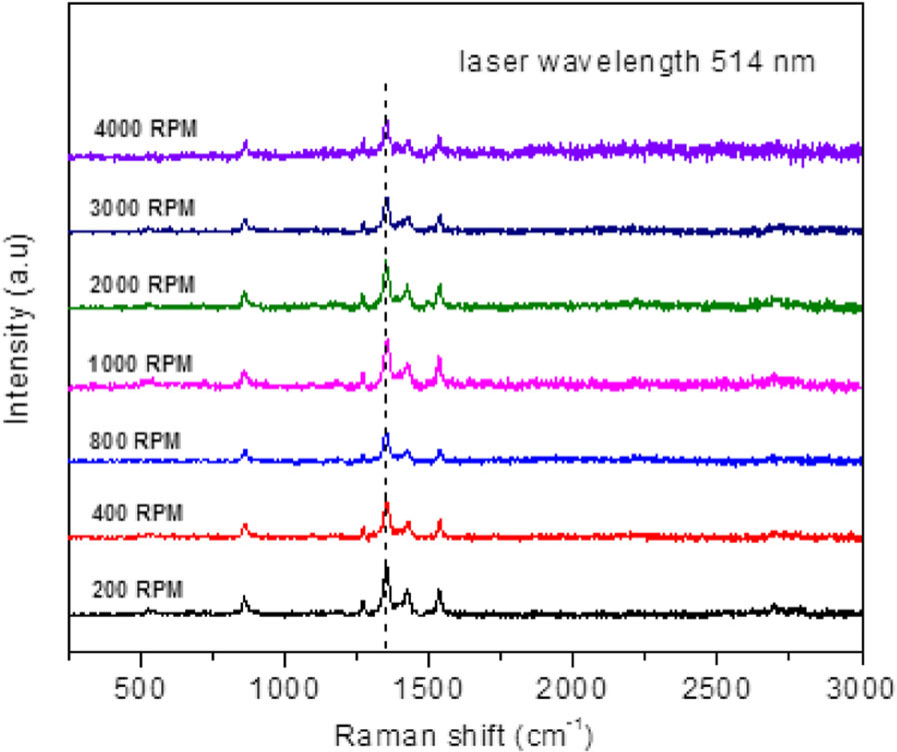
Raman spectra of PCPDTBT at different rotational speeds with excitation wavelength of 514 nm

By nanostructuring PCPDTBT and then varying the structures, slight changes have been observed in the peak positions of vibrational modes of the Raman shift (Fig. 10). The shifting usually takes place either towards the left (lower energy) or to the right position (higher energy). Table 2 shows peaks assigned to molecular vibrational modes of PCPDTBT. It also demonstrates that the vibration modes of PCPDTBT depend on the rotational speeds. All the rotational speeds show existence of seven modes in the Raman shift. The most intense peaks are recognized as the finger prints of PCPDTBT. The phonon modes that can be observed through the Raman measurement shows differentiation in response to different nanostructures which were recorded through dissimilar position of the Raman shift for the same vibrational mode assignments.

**Table 2:** Raman mode assignments and frequencies (cm^−1^)

Peak	200 rpm	400 rpm	800 rpm	1000 rpm	2000 rpm	3000 rpm	4000 rpm	Vibrational assignments
*v*_*1*_	526	524	526	528	534	527	535	C-C=O in-plane deformation
*v*_*2*_	861	861	862	858	861	861	863	CPDT: ring “breathing” (cyclopentane)
*v*_*3*_	1269	1272	1270	1272	1269	1273	1272	BT: C-H deformation (symmetric in-plane)
*v*_*4*_	1351	1351	1351	1354	1351	1349	1352	C-H deformation (isopropyl group)
*v*_*5*_	1423	1429	1427	1421	1426	1424	1429	CPDT: C-C stretch (dithiophenes)
*v*_*6*_	1535	1539	1539	1535	1537	1536	1535	BT: C-C stretch (in-plane C-H wag)
*v*_*7*_	2700	2702	2699	2695	2689	2711	2714	C-H stretching and bending

PCPDTBT conjugated polymer, comprising two units “CPDT” (electron-donating unit) and “BT” (electron-accepting unit), has shown strong polymer/fullerene interactions suggesting effective photoinduced charge transfer [20, 34]. Since the donating unit predominantly consists of C-H group, hence, from the Raman spectra, it can be seen that all the phonon modes of the Raman shift observed at about 2700 cm^−1^ correspond to C-H stretching and bending. The C-H deformation at 1350 cm^−1^ exhibits the most intense mode of vibration and is attributed to the isopropyl group of ethylhexyl which is coupled with the electron-donating unit (CPDT) to increase the solubility of the polymer [32]. The ring breathing of the CPDT was found at 863 cm^−1^ for the infiltration speed of 4000 rpm. The C-H deformation in the isopropyl group at 4000 rpm is shifted to higher energy. The C-H deformation of benzothiadiazole found at 1270 cm^−1^ shows a shift of 4 cm^−1^. The peak corresponding to C-C=O deformation absorbs energy at about 535 cm^−1^ for 4000 rpm and is shifted 6 cm^−1^ when compared with that of 200 rpm (~526 cm^−1^). The phonon mode of C-C stretch confirming cyclopentane in-plane stretching takes place at 1535 cm^−1^ which corresponds to the BT of the donor unit [34]. Again, a shift of 6 cm^−1^ is observed for the C-C stretch of CPDT at 1429 cm^−1^ when the lowest and highest infiltration speeds are compared.

## Conclusions

The aim of this work is to produce nanostructures of PCPDTBT conjugated copolymer through using the template-assisted method. A new technique to infiltrate solution into the nanochannels of the alumina template has been conceived by modifying the centrifuge tube for the centrifugation process. The produced nanostructures have revealed a strong correlation between the amount of centrifuge force and formation of nanostructures. Different infiltration forces obtained by varying rotational speeds produced diverse forms of nanostructures and their distribution. Fabricated nanostructures revealed multiple morphological, optical, and structural properties. The present study opens doors to fabricate desired nanostructures such as nanotubes, nanorods, and nanoflowers by controlling proper solution concentration and corresponding adhesive forces.

## Abbreviations

EDX: Energy-dispersive X-ray spectroscope
FESEM: Field emission scanning electron microscopy
NaOH: Sodium hydroxide
PCPDTBT: Poly{[4,4-bis(2-ethylhexyl)-cyclopenta-(2,1-*b*;3,4-*b*′)dithiophen]-2,6-diyl-*alt*-(2,1,3-benzothiadiazole)−4,7-diyl}
PL: Photoluminescence
